# Linoleic Acid Enhances Renal Tubular Epithelial Cells Autophagy Caused by Calcium Oxalate Monohydrate Crystals

**DOI:** 10.1111/jcmm.70250

**Published:** 2024-12-26

**Authors:** Weiming Ma, Wanqing Wei, Yang Dong, Yan Zhao, Tian Xia, Xitao Wang, Conghui Han

**Affiliations:** ^1^ Suzhou Medical College of Soochow University Suzhou, Jiangsu China; ^2^ Department of Urology Xuzhou Central Hospital Xuzhou, Jiangsu China; ^3^ Department of Urology Lianshui People's Hospital of Kangda College Affiliated to Nanjing Medical University Nanjing, Jiangsu China

**Keywords:** apoptosis, calcium oxalate kidney stones, linoleic acid, MFGE8, ROS

## Abstract

High intake of dietary linoleic acid may increase the incidence of many diseases. The aim of this research is to examine the impact of linoleic acid on the damage caused by calcium oxalate kidney stones on renal tubular epithelial cells. Calcium oxalate monohydrate (COM) crystals were prepared and used to treat HK‐2 cells, which were further treated with different concentrations of linoleic acid in vitro. Also, a small‐interfering RNA lentiviral vector of milk fat globule epidermal growth factor 8 (MFGE8) was constructed and transfected into HK‐2 cells. The cell viability, level of intracellular ROS and autophagy were tested. In vivo experiments were also carried out with a rat model for renal urolithiasis treated with linoleic acid. The results indicated that COM crystals promoted crystal deposition and apoptosis, increased levels of intracellular Ca^2+^ and ROS levels and inhibited the proliferation of HK‐2 cells. Linoleic acid exacerbated the damage of COM crystal‐treated HK‐2 cells and renal tubular epithelial cells of the rat model for renal urolithiasis, which can be partially reversed by downregulation of MFGE8. These results collectively suggest that linoleic acid might enhance the damage of renal tubular epithelial cells caused by COM crystals.

**Abbreviations**
COM: calcium oxalate monohydrate
ELISA: enzyme‐ linked immunosorbent assay
IHC: immunohistochemistry
LA: linoleic acid
ROS: reactive oxygen species

## 1 Introduction

Nephrolithiasis, a kidney stone disease, is highly prevalent, increasingly common and characterised by painful stone events that cause considerable morbidity. In the United States, about 1 out of every 11 people suffer from kidney stone disease, and 50% of patients will relapse [1]. Calcium oxalate (CaOx) kidney stone is the most prevalent type and has a high recurrence rate. Evidence suggests that a slight increase in urinary oxalate concentration could be a significant factor in CaOx urolithiasis [2]. Hyperoxaluria can be classified into three different forms: idiopathic, intestinal and primary. Among them, intestinal hyperoxaluria is caused by absorption disorders due to surgery (such as small bowel resection or weight loss surgery) or inflammatory bowel disease, which leads to increased oxalate absorption [3]. Currently, there are no specific treatments that are satisfactory for hyperoxaluria. The fundamental problem cannot be solved through surgical treatment. Dietary restriction is typically advised, but there are numerous foods that contain oxalate, making it challenging to stick to a low‐oxalate diet [4].

Linoleic acid (LA), an ω‐6 fatty acid, is the most prevalent polyunsaturated fatty acid in the human diet, which is crucial for numerous biological processes [5]. LA is usually promoted in the diet as an essential fatty acid beneficial to human health, but more and more evidence show that the excessive intake of LA will lead to the formation of oxidised linoleic acid metabolites (OXLAMs), which will result in the impairment of mitochondrial function through the unsatisfactory composition of cardiolipin [6, 7] and may induce many chronic diseases, such as cardiovascular diseases (CVDs) [8], cancer [9], diabetes [9] and Alzheimer's disease [10]. Studies have shown that the main cause of atherosclerotic changes is OXLAMs [11]. 4‐Hydroxynonenal (4‐HNE), a form of OXLAM, decomposed from seed oil at a faster rate during the heating process, which is a mutagen and a biomarker of oxidative stress and also known to cause DNA damage [12]. The level of 4‐HNE can be measured easily, the increase of which is related to heart failure [13, 14].

It was believed in early research that autophagy was detrimental to the development of kidney stones [15, 16]. The protective concept of autophagy related to kidney stones was first introduced in 2020 [17]. Previous research has suggested that CaOx crystals can enhance the expression of LC3‐II and BECN1 in HK‐2 cells, as well as increase the number of GFP‐LC3 spots and autophagic vacuoles in a dose‐ and time‐dependent manner. It was further revealed that CaOx crystals induced autophagy partly by activating the reactive oxygen species (ROS) pathway in HK‐2 cells. The use of 3‐methyladenine or siRNA to inhibit autophagy was found to alleviate the damage of HK‐2 cells induced by CaOx crystals [18]. Up to now, the confirmed signal pathway to induce protective autophagy is the TFEB pathway. Torin1 is an autophagy enhancer with strong TFEB specificity [19]. The application of Torin1 to M‐1 cells co‐incubated with COM resulted in a significant upregulation of autophagy, leading to decreased damage to lysosomes and mitochondrial cells. The experimental results also show that this upregulation of autophagy is related to the improvement of inflammatory reactions and crystal adhesion [20]. This research was focused on studying the effects of LA on the damage caused by CaOx crystals in renal tubular epithelial cells and the potential regulatory mechanism.

## 2 Materials and Methods

### 2.1 Preparation of COM Crystals

COM crystals were prepared in accordance with the study of Singhto et al. [21]. Calcium chloride dihydrate (10 mM) was mixed with sodium oxalate (10 mM), and the mixture was incubated at 37°C overnight. After centrifuging at 3000 rpm for 5 min, COM crystals were harvested. The supernatant was discarded, and the crystals were resuspended in sterile double distilled water. After further centrifugation at 3000 rpm for 5 min, sterile double distilled water was discarded, and the crystals were washed two times and air‐dried at 37°C. Finally, COM crystals were added to complete RPMI1640 medium to prepare crystal suspensions at concentrations of 10, 50 and 100 μg/mL, respectively.

### 2.2 Cell Culture and Treatment

The renal tubular epithelial cell line (HK‐2), purchased from the Type Culture Collection of the Chinese Academy of Sciences (Shanghai, China), was maintained in RPMI‐1640 (Gibco; Grand Island, NY) supplemented with 10% fetal bovine serum (Gibco) and antibiotics containing 1% penicillin G/streptomycin (P/S) (Sigma‐Aldrich).

In each well of 6‐well culture plates (Corning Inc.), about 5 × 10^5^ cells/2 mL were seeded and maintained 24 h before treatment. The HK‐2 cells reached 70%–80% confluence before being exposed to COM crystals at different concentrations (10, 50 and 100 μg/mL) in a complete medium; after being incubated for 24 h, the cells and medium were separately collected for further analysis. Cells in the control groups were added with an equal volume of complete medium.

LA (HY‐N0729) was dissolved in 100% ethanol to prepare different concentrations of solutions (0.1, 1, 10, 50, 100, 200, 500 and 1000 μM). COM‐dependent cells were treated with different concentrations of LA for 24 h to determine IC20, IC30 and IC50 for further analysis.

### 2.3 Assessment of Proliferation and Apoptosis of Cells

The cell viability was detected with an MTT (Beyotime, C0009S) assay using a 96‐well plate (200 μL/well). After cells were treated with crystal suspensions at concentrations of 10, 50 and 100 μg/mL, the waste medium was removed, and 10 μL of MTT solution was added into each well for incubation for 4 h. Each well was given Formazan to continue incubating for another 4 h. A microplate reader was used to detect the absorbance value of the sample at 490 nm. Experiments were repeated three times, and data were represented as the mean of five replicate wells ±SD.

Cell apoptosis was measured by Annexin‐V‐FITC/PI (Thermo, 556547) staining flow cytometry, according to the manufacturer's instructions. The cells were washed with PBS and then resuspended with 200 μL of Binding Buffer and 10 μL of Annexin‐V‐FITC. The samples were protected from light at 4°C for 30 min. Then, 300 μL of binding buffer and 5 μL of PI were given for analysis by flow cytometry with excitation at 488 nm and emission measured at 560 nm.

### 2.4 Detection of Intracellular Ca^2+^

The fluorescent probe Fura‐2 AM (Beyotime, China, S1052) was used to detect the cytoplasmic Ca^2+^ levels. The COM‐treated cells were exposed to 5 μM Fura‐2 AM at 37°C for 30 min without light. The extracellular Fura‐2 AM was removed by washing the cells three times with PBS. The fluorescence intensity was assessed by confocal fluorescence microscopy.

### 2.5 Intracellular ROS Measurement Using Flow Cytometry and DHE Staining

Intracellular ROS levels were measured using DCFH‐DA (Beyotime, China, S0033S) as the fluorescent probe. The COM‐treated cells were exposed to 10 μM DCFH‐DA at 37°C for 20 min without light. Cells were resuspended in PBS after washing them three times with PBS. Flow cytometry was utilised to analyse the fluorescence intensity.

### 2.6 Plasmid Construction, Lentivirus Infection and Transfection

In accordance with shRNA primer design principles, three human shRNA sequences and negative control shRNA (shNC) were designed by Sangon Biotech (Shanghai, China) (Table S1) and cloned in pLV‐puro vector to endogenously downregulate MFGE8. DNA sequencing (Majorbio, Shanghai, China) was used to confirm the synthesised core plasmid. Lipofectamine 3000 (Invitrogen, Shanghai, China) was used to transfect the lentiviral vector and packaging plasmids into 293 T cells for lentiviral production. After 48 and 72 h of transfection, the culture media containing lentivirus were collected and filtered. Then, the indicated lentivirus was used to infect the HK‐2 cells. The medium was replenished after 24 h of infection, and the cells were put into a cell incubator to continue cultivation. Three days later, a complete medium with 2 μg/mL puromycin was used to select positive cells for subsequent experiments.

### 2.7 Quantitative Real‐Time Polymerase Chain Reaction (qRT‐PCR)

The manufacturer's protocol was followed for the extraction of total RNA using TRIzol reagent (Invitrogen, Carlsbad, CA). cDNA synthesis was carried out on each 2 μg RNA sample by means of the Reverse Transcriptase Kit (Thermo, USA). Then, qPCR was performed on a real‐time detector using a SYBR Green PCR kit (Thermo, USA). GAPDH was used as the internal reference for normalisation. The expression data were calculated using the $2^{-\Delta \Delta C_{t}}$ method. Primer sequences of MFGE8 were as follows: primer F, 5′‐CTCGTCTGTGCGTGTGACCTTC‐3′ and primer R, 5′‐TTATCGTCATTGCTGCTGGGTGTC‐3′.

### 2.8 Western Blotting (WB)

Cells were lysed using RIPA buffer (Millipore, Temecula, CA, USA) containing 1% protease inhibitor, and the total protein was collected. The BCA Protein Assay Kit (HyClone‐Pierce, Logan, UT, USA) was used to detect the concentration. Then, the protein sample (20 μg) was separated by 10% SDS‐PAGE gels and were transferred onto PVDF membranes. After blocking with 5% bovine serum albumin for 1 h at room temperature, the membranes were incubated with primary antibodies against MFGE8 (1:1000, Proteintech, USA), GSKIP (1:1000, Sangon, China), TCEA3 (1:1000, Proteintech, USA), MAP3K20 (1:1000, Proteintech, USA), OBI1 (1:1000, Proteintech, USA), LC3 (1:1000, Proteintech, USA), Beclin1(1:1000, Proteintech, USA) and GAPDH (1:2000, Cell Signalling, Germany) overnight at 4°C. Then, the membranes were incubated with HRP‐conjugated secondary goat antirabbit antibodies for 1 h at room temperature after three washes with TBST buffer. The protein bands were finally viewed by enhanced chemiluminescence. ImageJ was utilised to quantify the relative density of each band.

### 2.9 Protein Identification by LC–MS/MS

The high‐throughput proteomic analyses were carried out using LC–MS/MS. The default settings of Spectronaut X (Biognosys AG, Switzerland) were used to process the raw DIA data. Differentially expressed proteins were screened using Student's *t*‐test (*Q* value < 0.05, absolute AVG log_2_ ratio > 0.58).

### 2.10 Immunofluorescence

HK‐2 cells pretreated were transferred to 35‐mm dishes with a density of 30,000 cells per dish. On the day following, the cells were fixed with 4% paraformaldehyde and kept at room temperature for 20 min. Then, they were permeabilized with 0.5% TBS‐Triton plus 5% bovine serum albumin at room temperature for 30 min. The primary antibody (LC3, 1:500, Proteintech, USA; Beclin1, 1:200, Proteintech, USA) was incubated overnight at 4°C. After rewashing and permeabilization, the samples were incubated in the corresponding secondary antibodies for 1 h. Then, DAPI staining was performed for 5 min. The sealed slices were immediately observed using fluorescence microscopy after washing.

### 2.11 Animal Experiments

Thirty male SD rats (200–250 g) were purchased from Xipur‐Bikai Experimental Animal Co. Ltd. (Shanghai). All animal procedures were approved by the Animal Care and Use Committee of Xuzhou Central Hospital (Issue No: 202302T006). Experimental animals were euthanized using the cervical dislocation method. All rats were divided into three groups, with 10 rats in each group: control group, model group and model + LA group. The model groups received an aqueous solution containing 1% ethylene glycol (EG), while the control group received normal drinking water. After a week of feeding, the rats in the model + LA group received LA intraperitoneal injections daily for 4 weeks. Urine and blood of all rats were collected. At the end of the experimental procedures, all animals were euthanized, and the kidneys were harvested for further analysis.

### 2.12 Kidney Histologic Staining

The rats' kidney samples were fixed in 4% paraformaldehyde, embedded in paraffin and cut into thick slices. Von Kossa staining (Solarbio, Beijing, China) was applied to assess the deposition of crystals, according to the manufacturer's instructions. Meanwhile, the kidney tissue sections were also stained with haematoxylin and eosin (HE) to detect crystal deposition. Finally, they were observed and recorded under a microscope.

### 2.13 Immunohistochemistry

After paraffin‐embedded sections were dewaxed and dehydrated, they were subjected to repair the antigen with boiling citric acid buffer and block endogenous peroxidase with 3% H_2_O_2_. The sections were naturally cooled, washed and incubated with MFGE8 antibody at 4°C overnight and HRP‐conjugated secondary antibody (Thermo Fisher, USA, 1:500) at 37°C for 1 h, followed by incubation with DAB solution. Re‐staining, differentiation, dehydration and blocking processes were performed for the sections. The microscope was used for the acquisition of all images.

### 2.14 Enzyme Linked Immunosorbent Assay

The concentrations of H_2_O_2_, LDH and MDA in the urine of rats were determined using ELISA kits (Leagene, China) following the protocols. A microplate reader was utilised to detect the absorbance value at 450 nm.

### 2.15 Statistical Analysis

The study protocols were approved by the Ethical Committee Review Board of the Xuzhou Central Hospital (Xuzhou, Jiangsu, China) (No. 202302T006). GraphPad Prism (GraphPad Software, version 8.0.1, La Jolla, CA, USA) software was used for data analysis. Three or more independent experiments were conducted, and the result was expressed as the mean ± standard deviation (SD). Two group comparisons were performed using an unpaired *t*‐test, and multiple group comparisons were carried out using one‐way analysis of variance (ANOVA). The *p*‐values < 0.05 were considered statistically significant.

## 3 Results

### 3.1 Impact of COM Crystals on Morphology, Proliferation, Apoptosis and Oxidative Stress in HK‐2 Cells

**FIGURE 1:**
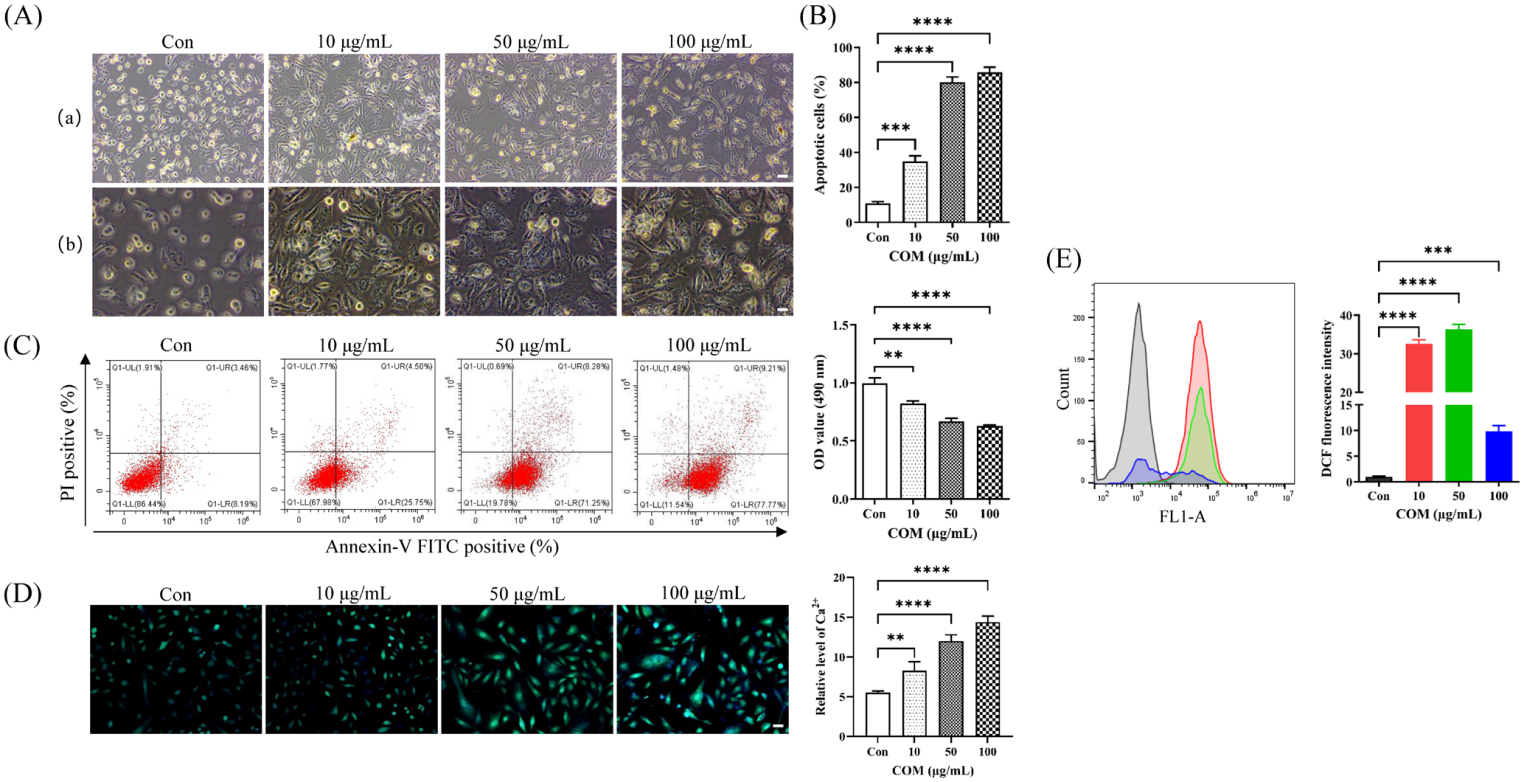
Damage caused by calcium oxalate (CaOx) kidney stone to renal tubular epithelial cells. (A) Construction of cell models of calcium oxalate (CaOx) kidney stone induced by COM crystals. Different magnifications showed the deposition of crystals. With increased concentration of COM crystals, more crystals are deposited in the cytoplasm. (B) The MTT assay demonstrated the proliferation activity of HK‐2 cells in different concentrations of COM crystals. **p = 0.0002, ****p < 0.0001. (C) Different concentrations of COM crystals affected the degree of apoptosis of HK‐2 cells. ****p < 0.0001. (D, E) Intracellular Ca^2+^ levels and ROS in HK‐2 cells after treatment with different concentrations of COM crystals, respectively. (D) **p = 0.0071, ****p < 0.0001; (E) ***p = 0.0007, ****p < 0.0001.

A concentration gradient of COM crystals (10, 50 and 100 μg/mL) was set to investigate its influence on morphology, proliferation, apoptosis and oxidative stress in human proximal tubule epithelial cells HK‐2. With the increase of COM crystal concentration, a significant morphological change occurred in HK‐2 cells with a large amount of crystal deposition in the cytoplasm (Figure 1A). The proliferation activity of HK‐2 cells was decreased (Figure 1B), and the proportion of apoptosis was escalated (Figure 1C). Fura‐2 AM showed a gradual rise in intracellular Ca^2+^ in HK‐2 cells (Figure 1D), and there was a significant rise in intracellular ROS levels (Figure 1E), inhibition, apoptosis and an increase of intracellular Ca^2+^ and ROS levels of HK‐2 cells.

### 3.2 Linoleic Acid Aggravated the Apoptosis and Autophagy Caused by CaOx Kidney Stones in Renal Tubular Epithelial Cells

**FIGURE 2:**
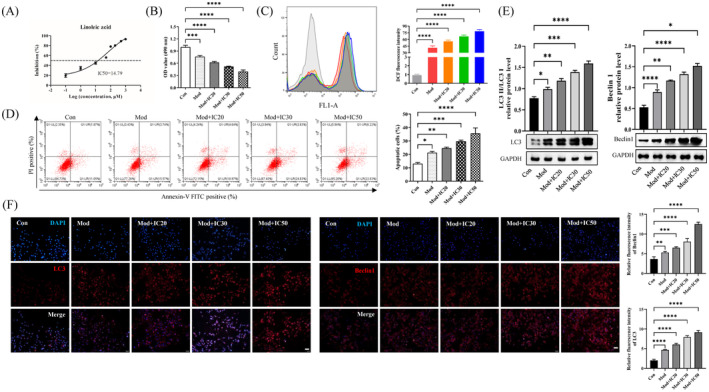
Renal tubular epithelial cells were more susceptible to injury due to CaOx kidney stones when linoleic acid was present. (A) Determination of IC50 of linoleic acid. (B) COM crystal‐treated HK‐2 cells were tested for proliferation activity following the addition of various levels of linoleic acid. **p = 0.0022, ****p < 0.0001. (C, D) Different concentrations of linoleic acid were added to COM crystal‐treated HK‐2 cells, resulting in changes in intracellular ROS levels and apoptosis. (C) ***p = 0.0006, ****p < 0.0001; (D) *p = 0.0435, **p = 0.0061, ***p = 0.0004, ****p < 0.0001. (E) The expression of autophagy biomarkers (LC3 and Berlin‐1) was altered by the addition of different concentrations of linoleic acid to COM crystal‐treated HK‐2 cells. *p = 0.0292, **p = 0.0016, ***p = 0.0002, ****p < 0.0001 for LC3; *p = 0.0110, **p = 0.0083, ***p = 0.0002, ****p < 0.0001 for Beclin1. (F) The results from immunofluorescence further illustrated that the expression of autophagy biomarkers (LC3 and Berlin‐1) was altered by the addition of different concentrations of linoleic acid to COM crystal‐treated HK‐2 cells. **p = 0.0093, ***p = 0.0002, ****p < 0.0001.

Firstly, we explored the dose effect of LA on the proliferation of HK‐2 cells (Figure 2A). The IC50 was confirmed to be 14.79 μM. In this part of the experiment, HK‐2 cells were treated with 10 μg/mL COM crystals to construct an in vitro cell model. Then, different concentrations of LA (IC20, IC30 and IC50) were subjected to COM crystal‐treated HK‐2 cells. As the concentration of LA increased, the proliferation activity of COM crystal‐treated HK‐2 cells was decreased (Figure 2B). Incrementally higher intracellular ROS levels and apoptosis were also observed in the treated cells (Figure 2C,D). Surprisingly, we found that the autophagy‐associated proteins were increased accompanied by the LA treatment. LC3‐II and Beclin1 were commonly regarded as autophagy markers [22]. A higher concentration of LA induced the expression of LC3‐II and Beclin1, implicating an increased autophagy level (Figure 2E). Immunofluorescence analysis of LC3 and Beclin1 further confirmed the results (Figure 2F). These findings suggested that LA aggravated apoptosis, intracellular ROS levels and autophagy stimulated by COM crystals in HK‐2 cells.

### 3.3 Alteration of Protein Profiles in Linoleic Acid‐Treated KS Model Cells

**FIGURE 3:**
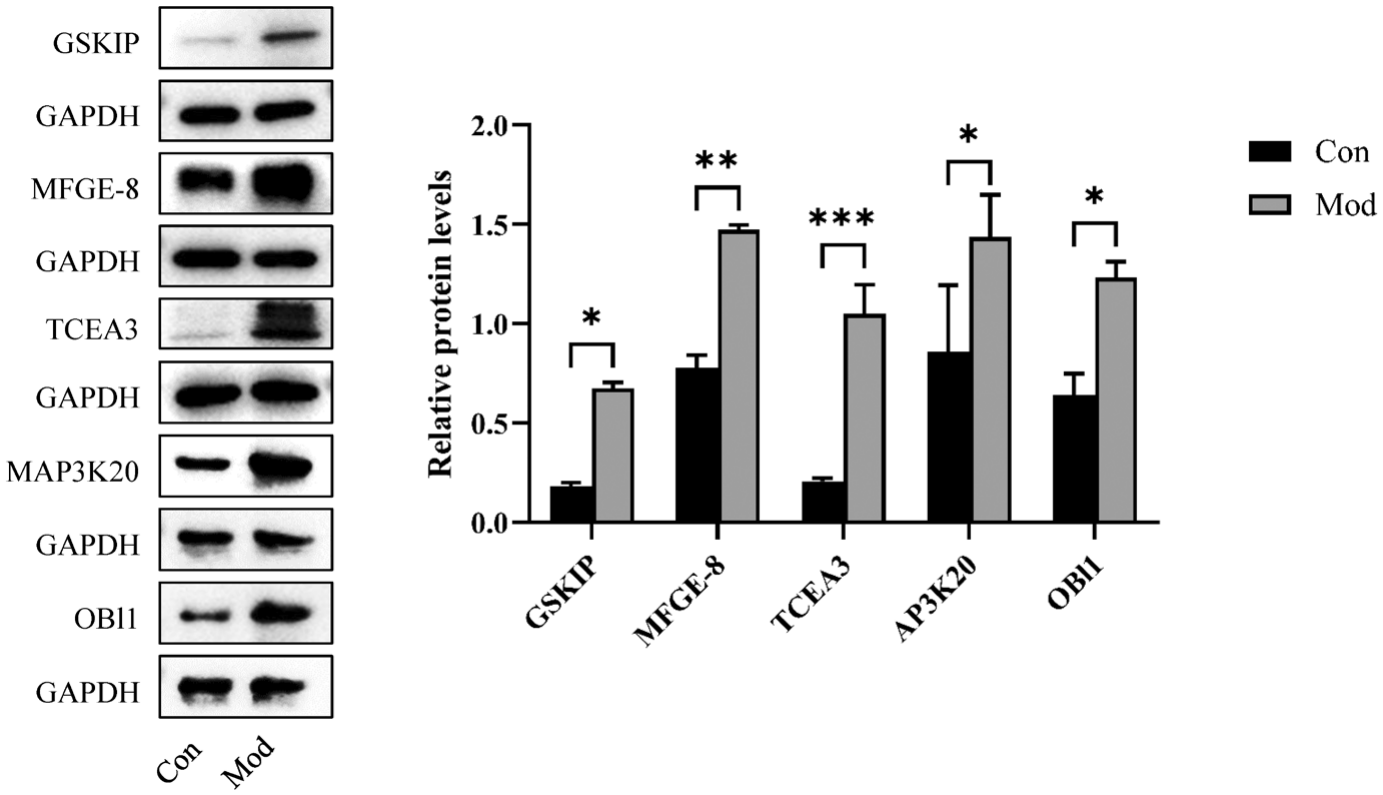
Differential expression protein screening. LC–MS/MS was performed between the model group and the model + linoleic acid group to identify differentially expressed proteins (MAP3K20, TCEA3, MFGE8, OBl1 and GSKIP), which were tested by WB. *p = 0.0302 for GSKIP, **p = 0.0033 for MFGE‐8, ***p = 0.0008 for TCEA3, *p = 0.0118 for MAP3K20, *p = 0.0103 for OBl1.

In order to investigate the potential regulatory mechanism for LA activating autophagy in COM crystal‐treated HK‐2 cells, the alteration of protein profiles was determined by LC–MS/MS. According to the screening criteria, we selected multiple proteins that had significant relative changes for subsequent analysis. MAP3K20, TCEA3, MFGE8, OBl1 and GSKIP were selected, and they were highly expressed in the model+IC50 group (Figure 3). According to the references, MFGE8 was related to autophagy in early brain injury (PMID: 38095841) and pancreatic fibrosis (PMID: 34421598); however, whether it is associated with autophagy aggravated by LA in kidney stones remains unknown.

### 3.4 Downregulation of MFGE8 Protected Against Linoleic Acid‐Inducing Injury in COM Crystal‐Treated HK‐2 Cells

**FIGURE 4:**
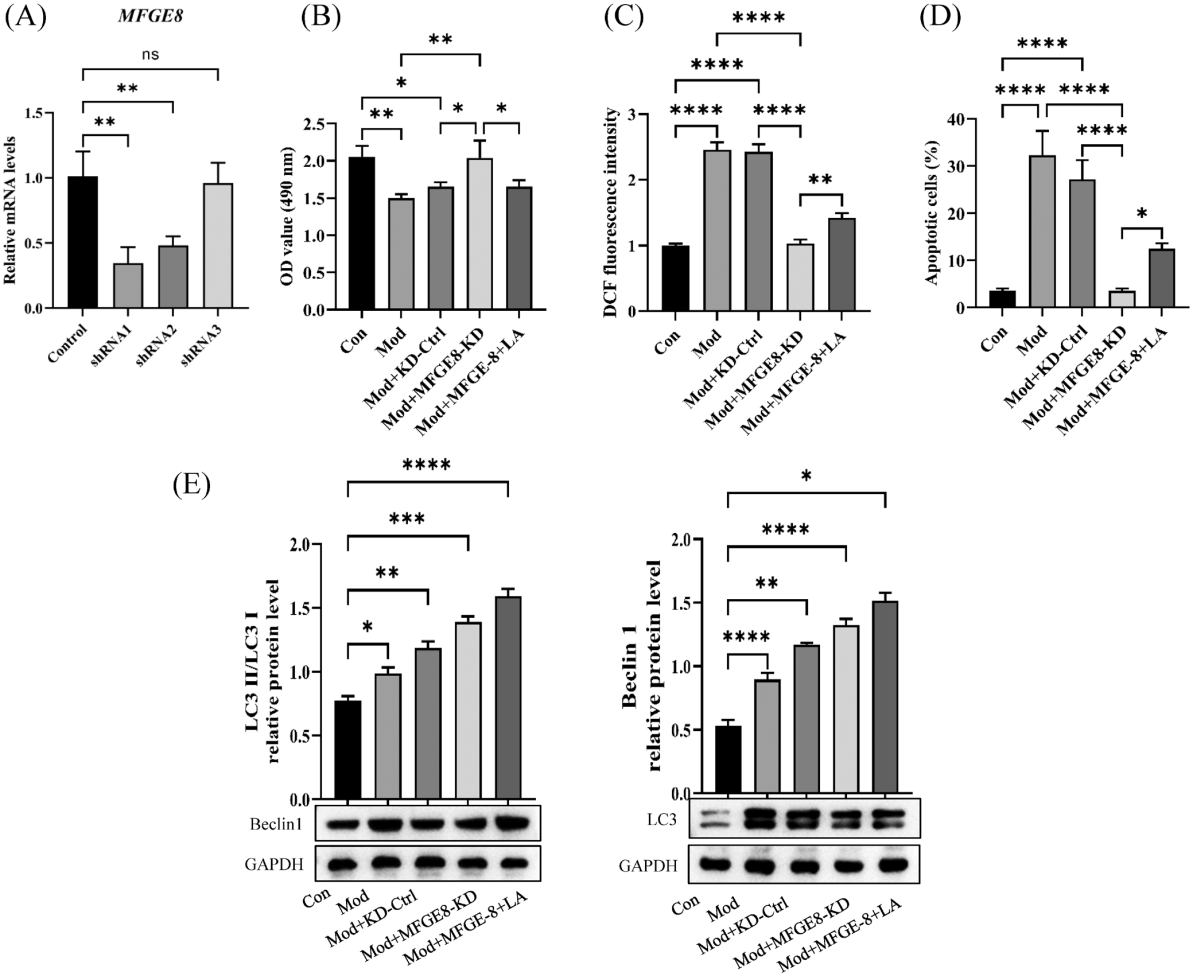
Downregulation of MFGE8 protected against linoleic acid causing damage to COM crystal‐treated HK‐2 cells. (A) Determination of knockdown efficacy of three human shRNAs for MFGE8. **p = 0.0012 for shRNA1, **p = 0.0048 for shRNA2. shRNA1 was selected for further analysis. (B) The proliferation activity of COM crystal‐treated HK‐2 cells with MFGE8 knockdown was evaluated. The viability of proliferation was observed to decrease after being treated with linoleic acid. **p = 0.0034 for Con vs. Mod, *p = 0.0272 for Con vs. Mod+KD‐Ctrl, *p = 0.0269 for Con vs. Mod+MFGE‐8+LA, **p = 0.043 for Mod vs. Mod+MFGE‐8‐KD, *p = 0.0348 for Mod+KD‐Ctrl vs. Mod+MFGE‐8‐KD, *p = 0.0343 for Mod+MFGE‐8‐KD vs. Mod+MFGE‐8+LA. (C–E) Downregulation of MFGE8 inhibited apoptosis, ROS accumulation and autophagy of COM crystal‐treated HK‐2 cells. Linoleic acid promoted apoptosis, ROS accumulation and autophagy in MFGE8‐downregulated COM crystal‐treated HK‐2 cells. (C) **p = 0.0013, ***p = 0.0007, ****p < 0.0001; (D) *p = 0.0296, ****p < 0.0001.

To further investigate the effect of MFGE8, three independent shRNAs of MFGE8 were designed and packaged as lentiviruses to infect HK‐2 cells, and the most effective target, shRNA‐1, was selected for subsequent experiments (Figure 4A). The result showed that MFGE8 knockdown increased the proliferation viability of COM crystal‐treated HK‐2 cells compared with the control group. The viability of proliferation was decreased after being treated with LA (Figure 4B). On the other hand, downregulation of MFGE8 inhibited apoptosis, ROS accumulation and autophagy of COM crystal‐treated HK‐2 cells, compared with the control group (Figures 4C–E and S1). However, these biological statuses were partially reversed by LA, which promoted apoptosis, ROS accumulation and autophagy in MFGE8‐downregulated COM crystal‐treated HK‐2 cells. These results indicated that downregulation of MFGE8 protected against LA, stimulating apoptosis, increasing intracellular ROS levels and inducing autophagy in COM crystal‐treated HK‐2 cells.

### 3.5 Linoleic Acid Enhanced the Damage Caused by COM Crystals to Renal Tubular Epithelial Cells In Vivo

**FIGURE 5:**
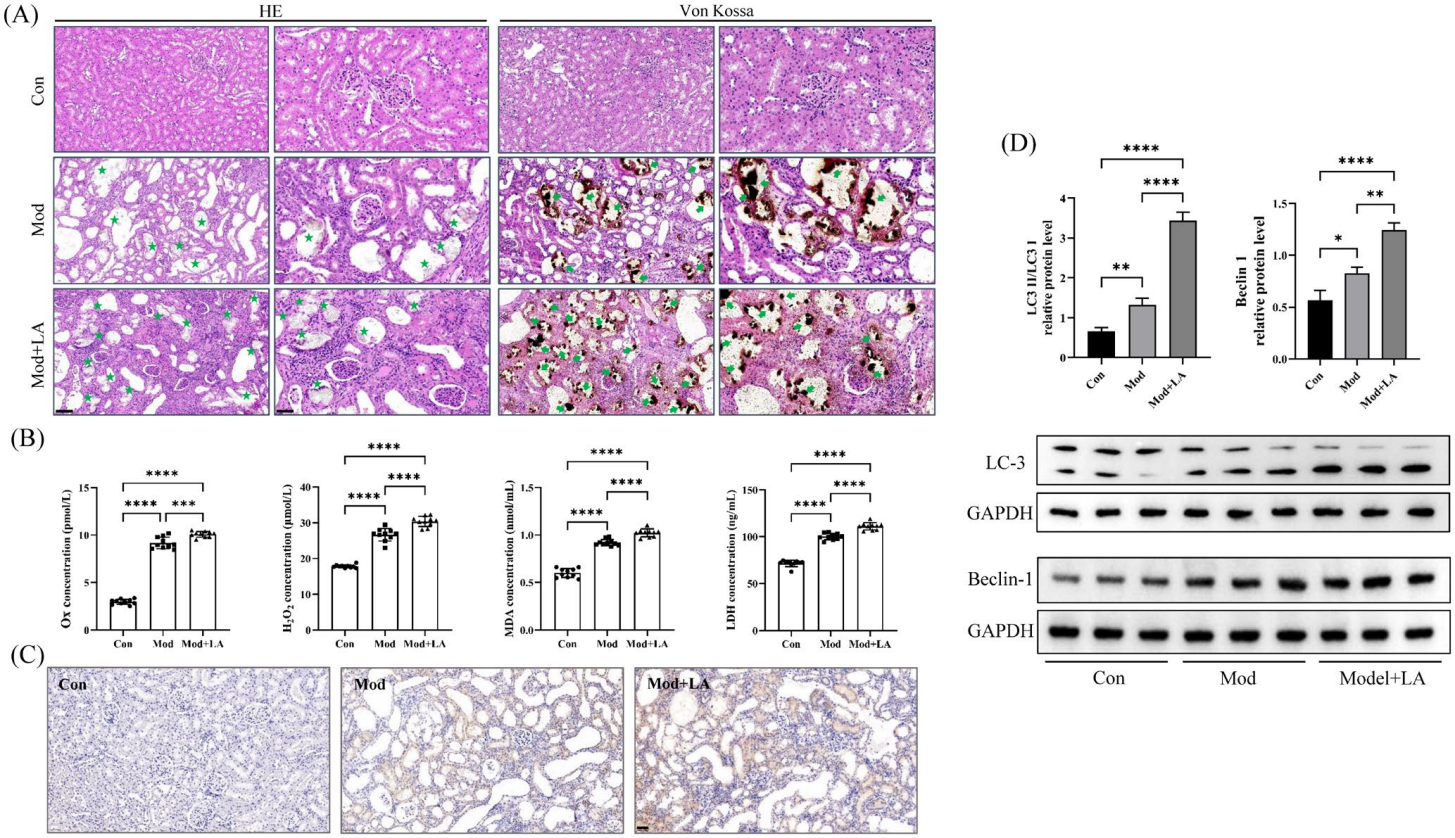
The damage caused by COM crystals to renal tubular epithelial cells in vivo was increased by linoleic acid. (A) The difference in dilated and atrophied kidney lumen and transparent calcium oxalate stone crystal deposits in each group of rats was shown by HE staining and Von Kossa staining. (B) The level of oxalic acid, H_2_O_2_, LDH and MDA in urine was tested in each group of rats by ELISA. **P = 0.0022, ****P < 0.0001. (C) The expression of MFGE8 in kidney tissue in each group of rats was tested by immunohistochemistry. ***P = 0.0006, ****P < 0.0001;. (D) The expression of autophagy biomarkers, LC3 and Beclin1, in kidney tissue in each group of rats was tested by WB. *P = 0.0435, **P=0.0061, ***P=0.0004, ****P < 0.0001.

Firstly, as illustrated by H&E staining, the model group and the model+LA group showed clear dilated and atrophied kidney lumen in rats, with a significant amount of transparent calcium oxalate stone crystal deposits (black arrow) compared to the control group (Figure 5A). Further, Von Kossa staining indicated that the model+LA group displayed a higher level of calcium oxalate stone crystal deposits (Black arrow) and renal tissue damage, compared to the model group. Through urine analysis, the model+LA group had an elevated level of oxalic acid, H_2_O_2_, LDH and MDA compared to the model group and the control group, as indicated by the ELISA results (Figure 5B). The kidney tissue of rats in the model group showed a high expression of MFGE8, which was further increased after treatment with LA (Figure 5C). High expression was seen in the model group for the autophagy biomarkers LC3 and Beclin1 (Figure 5D). The model+LA group showed a clear trend. We concluded that the damage caused by COM crystals to renal tubular epithelial cells was made worse by LA in vivo.

## 4 Discussion

The assessment of nutritional risk factors is an essential component in investigating the formation and prevention of kidney stones. By implementing an appropriate dietary approach, the effective prevention of recurring stones can be achieved, and the burden of invasive surgical procedures can be reduced for the treatment of urinary stone disease. LA has been deemed an essential fatty acid, and an excessive intake of it has been linked to numerous chronic diseases, including obesity and diabetes [5]. However, the effect of LA on kidney stones remains unclear. In the study, we focused on the role of LA in the damage that CaOx kidney stones cause to renal tubular epithelial cells. We discovered that LA magnified the harm that CaOx kidney stones brought to renal tubular epithelial cells, including stimulating apoptosis, increasing intracellular ROS and Ca^2+^ levels and inducing autophagy in vitro and in vivo. Further analysis demonstrated that these biological processes may be partially reversed by the downregulation of MFGE8.

Incorporating inorganic substances (such as crystalline salts) and organic components (such as urinary macromolecules) within the renal parenchyma or pelvicalyceal system causes the creation of kidney stones, which serves as the pathological basis for the formation of kidney stones. Calcium oxalate stones are the most prevalent type and can result in several injuries to renal tubular epithelial cells, including releasing intracellular Ca^2+^ [23], damaging mitochondria to increase ROS and aggravate the inflammatory response [24] and inducing cell death (such as apoptosis, ferroptosis, autophagy and pyroptosis) [25, 26, 27, 28]. Based on the above characteristics, we have successfully constructed a model for CaOx kidney stones. CaOx crystals triggered AT1R to promote Ca^2+^ release medicated by IP3/IP3R, resulting in an increase in cytoplasmic Ca^2+^ levels [23]. Ca^2+^, which is an important second messenger, is responsible for regulating most signalling pathways in both physiological and pathological processes. ROS production depends on Ca^2+^ signalling, and an increase in intracellular Ca^2+^ can trigger ROS‐generating enzymes to cause oxidative stress [29]. During oxidative stress (pathological condition), ROS‐induced ROS release is caused by the mitochondrial scavenging system being overwhelmed by the perpetual increase in ROS production [30]. This leads to changes in mitochondria's functions, which in turn cause mtDNA harm and oxidative changes to mitochondrial proteins and enzymes in the TCA cycle and ETC [30]. These changes can trigger the release of proinflammatory cytokines and initiate the inflammatory process [31]. In addition, cells undergo apoptosis due to mitochondrial dysfunction, which can cause cell shrinkage, plasma membrane blebbing, organelle condensation and fragmentation. Thus, the tubular lumen becomes populated with oxalate and CaOx crystals after the release of apoptotic bodies, cell debris and fragmented subcellular organelles, which serve as the raw materials for the stone nidus formation [32]. Previous studies have demonstrated that various cell deaths can be a result of CaOx crystals. The ERS‐ROS‐NF‐κB signalling pathway in renal tubular epithelial cells was activated to induce apoptosis [25]. Ferroptosis mediated by the p53/SLC7A11 pathway is activated by ANKRD1 to initiate and develop CaOx kidney stones [26]. The SIRT1‐mediated signalling pathway is activated to regulate CaOx crystal‐induced autophagy [27]. Chen et al. have discovered that the NLRP3‐GSDMD pathway was involved in the oxalate‐induced pyroptotic injury in HK‐2 cells [28]. It can be deduced from the results that the damage caused by calcium oxalate stones to renal tubular epithelial cells is both multifaceted and complex.

LA is found mostly in vegetable oils, nuts, seeds, meats and eggs in the diet, with its intake increasing due to changes in the modern diet structure. It has been proposed that the high intake of dietary LA may increase the incidence of chronic diseases, such as CVD, cancer and inflammation. The potential mechanism for these negative health outcomes is based on the transformation of LA into arachidonic acid and its subsequent eicosanoids derived thereof. Baggio et al. [33] have found that plasma contains less LA and more arachidonic acid, and the arachidonic acid/LA ratio was higher in individuals with idiopathic calcium stones than in healthy controls. Messa et al. [34] have also found higher levels of arachidonic acid in hyperoxaluric stone‐formers than in stone‐formers with normal oxalate excretion. However, the investigation of the potential mechanism for the effect of higher levels of LA on renal tubular epithelial cells is still ongoing. In our study, we reported that LA enhanced the damage caused by COM crystals to renal tubular epithelial cells in vivo and in vitro. Proteomics analysis showed that this process was regulated by MFGE8. The phagocytosis of apoptotic cells is mediated by MFGE8 due to its epidermal growth factor (EGF)‐like domains at the N‐terminal and F5/8‐Type C domains at the C‐terminal [35]. In the central nervous system, MFGE8 alleviates neuronal cell apoptosis and neuroinflammation through various pathways when brain injury occurs [36, 37]. The downstream central regulatory molecules include integrin β3 and HMGB1. In addition, another mechanism for protecting neuronal cells is to regulate the polarisation of microglia. MFGE8 was also discovered to be involved in the regulation of lung injury by mitigating neutrophil infiltration [38]. However, there are few studies to reveal the role of MFGE8 in the modulation of kidney stones. Our study found that downregulation of MFGE8 protected against LA, stimulating apoptosis, increasing intracellular ROS levels and inducing autophagy in COM crystal‐treated HK‐2 cells. Further research is needed on the potential signalling pathway. Although we showed that the role of LA in vivo being consistent with that in vitro, due to intestinal metabolism processing LA, its direct effect on exacerbated damage to renal tubular epithelial cells was complicated. Further investigation is necessary to identify the metabolites of LA that are being involved.

## 5 Conclusion

In summary, we demonstrated that LA enhanced the damage caused by COM crystals to renal tubular epithelial cells by stimulating apoptosis, increasing intracellular ROS levels and inducing autophagy, which will be partially reversed by regulation of MFGE8. Based on our findings, stone prevention can be achieved through diet control, particularly a high‐fat diet.

## Author Contributions

**Weiming Ma:** conceptualization (equal), data curation (equal), formal analysis (equal), funding acquisition (equal), methodology (equal), software (equal), validation (equal), writing – original draft (lead), writing – review and editing (lead). **Wanqing Wei:** conceptualization (equal), data curation (equal), software (equal). **Yang Dong:** data curation (equal), methodology (equal), software (equal). **Yan Zhao:** data curation (equal), methodology (equal), software (equal), writing – review and editing (equal). **Tian Xia:** data curation (equal), software (equal). **Xitao Wang:** data curation (equal), software (equal). **Conghui Han:** conceptualization (equal), project administration (equal).

## Ethics Statement

The study protocols were approved by the Ethical Committee Review Board of the Xuzhou Central Hospital (Xuzhou, Jiangsu, China) (No. 202302T006).

## Consent

The authors have nothing to report.

## Conflicts of Interest

The authors declare no conflicts of interest.

## Supporting information


Figures S1–S5.

Table S1.

## Data Availability

The data generated in this article can be shared and obtained by contacting the corresponding author.
