# Supplementary figures and images for: Linoleic Acid Enhances Renal Tubular Epithelial Cells Autophagy Caused by Calcium Oxalate Monohydrate Crystals
Source: J Cell Mol Med. 2024 Dec 26;28(24):e70250. doi: 10.1111/jcmm.70250 (PMC11671237; doi:10.1111/jcmm.70250)

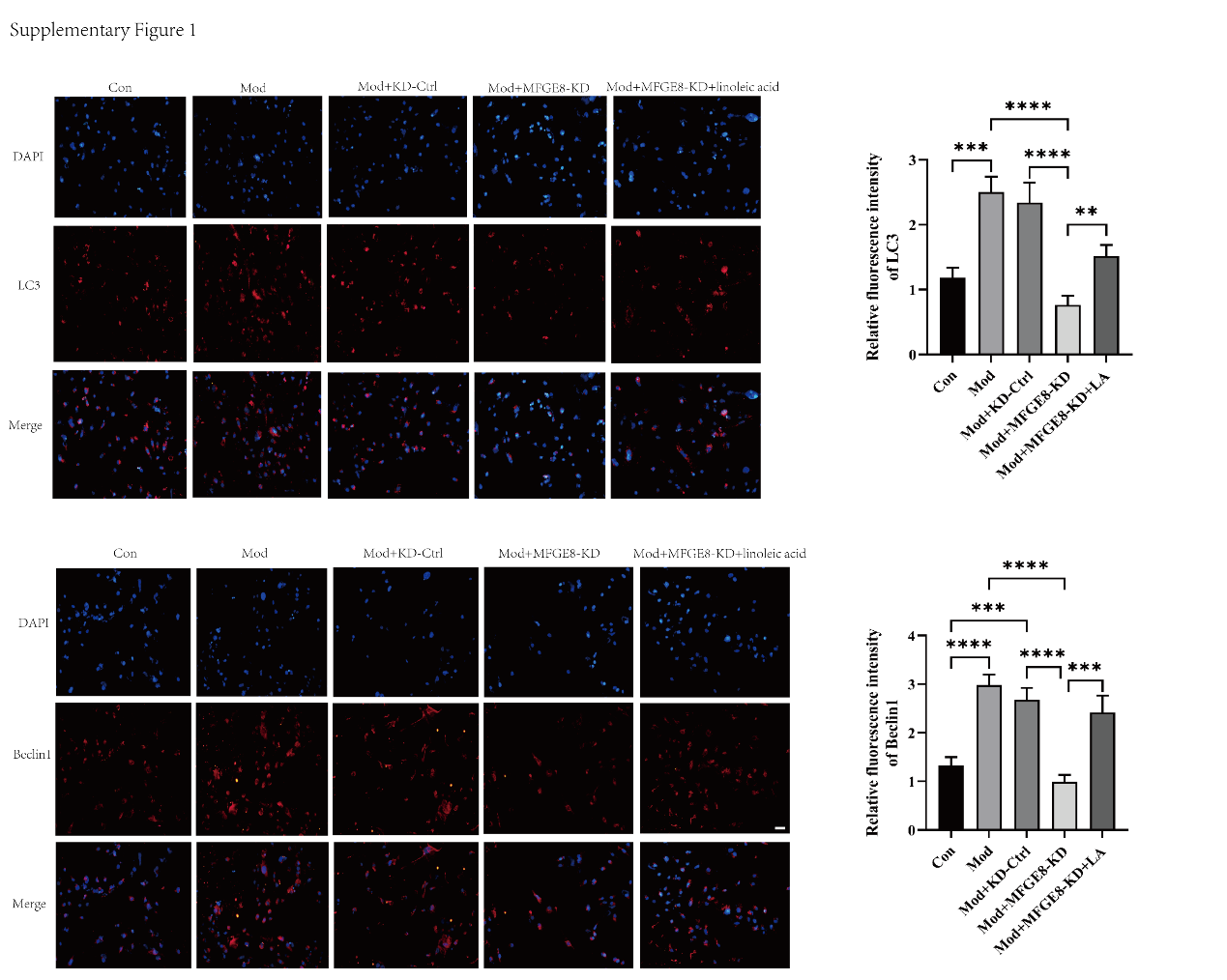


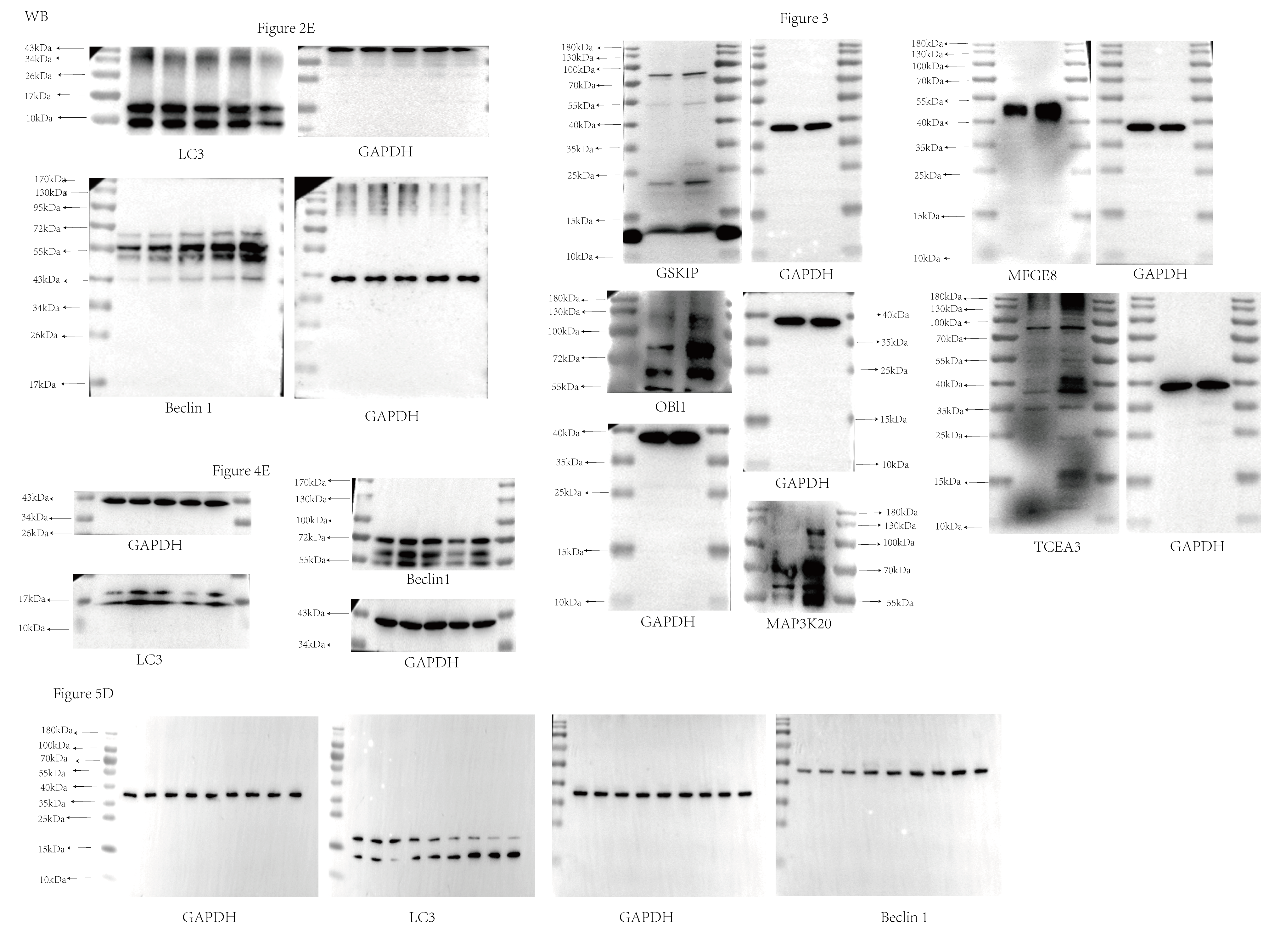

Supplement: Supplementary file 1 — Figures S1–S5. [file JCMM-28-e70250-s002.docx]
