# Supplementary material for: Linoleic Acid Enhances Renal Tubular Epithelial Cells Autophagy Caused by Calcium Oxalate Monohydrate Crystals
Source: J Cell Mol Med. 2024 Dec 26;28(24):e70250. doi: 10.1111/jcmm.70250 (PMC11671237; doi:10.1111/jcmm.70250)
Supplement: Supplementary file 2 — Table S1. [file JCMM-28-e70250-s001.docx]

Supplementary Table 1: Three shRNA sequences for MFGE8

Primer Sequence (5’ to 3’)

| MFGE8-shRNA1-F | GATCCgcaaccactgtgagacgaaatCTCGAGatttcgtctcacagtggttgcTTTTTG |
| --- | --- |
| MFGE8-shRNA1-R | AATTCAAAAAgcaaccactgtgagacgaaatCTCGAGatttcgtctcacagtggttgcG |
| MFGE8-shRNA2-F | GATCCcggtggtttatgcgaggagatCTCGAGatctcctcgcataaaccaccgTTTTTG |
| MFGE8-shRNA2-R | AATTCAAAAAcggtggtttatgcgaggagatCTCGAGatctcctcgcataaaccaccgG |
| MFGE8-shRNA3-F | GATCCacccagcagcaatgacgataaCTCGAGttatcgtcattgctgctgggtTTTTTG |
| MFGE8-shRNA3-R | AATTCAAAAAacccagcagcaatgacgataaCTCGAGttatcgtcattgctgctgggtG |
